# Candidate Therapeutics by Screening for Multitargeting Ligands: Combining the CB2 Receptor With CB1, PPARγ and 5-HT4 Receptors

**DOI:** 10.3389/fphar.2022.812745

**Published:** 2022-02-28

**Authors:** Shayma El-Atawneh, Amiram Goldblum

**Affiliations:** Molecular Modelling and Drug Design Lab, Institute for Drug Research and Fraunhofer Project Center for Drug Discovery and Delivery, Faculty of Medicine, The Hebrew University of Jerusalem, Jerusalem, Israel

**Keywords:** cannabinoid receptors 2 (CB2R), multitargeting, ISE, virtual screening, inflammation, neuroprotective, IBD—inflammatory bowel diseases

## Abstract

In recent years, the cannabinoid type 2 receptor (CB2R) has become a major target for treating many disease conditions. The old therapeutic paradigm of “one disease-one target-one drug” is being transformed to “complex disease-many targets-one drug.” Multitargeting, therefore, attracts much attention as a promising approach. We thus focus on designing single multitargeting agents (MTAs), which have many advantages over combined therapies. Using our ligand-based approach, the “Iterative Stochastic Elimination” (ISE) algorithm, we produce activity models of agonists and antagonists for desired therapeutic targets and anti-targets. These models are used for sequential virtual screening and scoring large libraries of molecules in order to pick top-scored candidates for testing *in vitro* and *in vivo*. In this study, we built activity models for CB2R and other targets for combinations that could be used for several indications. Those additional targets are the cannabinoid 1 receptor (CB1R), peroxisome proliferator-activated receptor gamma (PPARγ), and 5-Hydroxytryptamine receptor 4 (5-HT4R). All these models have high statistical parameters and are reliable. Many more CB2R/CBIR agonists were found than combined CB2R agonists with CB1R antagonist activity (by 200 fold). CB2R agonism combined with PPARγ or 5-HT4R agonist activity may be used for treating Inflammatory Bowel Disease (IBD). Combining CB2R agonism with 5-HT4R generates more candidates (14,008) than combining CB2R agonism with agonists for the nuclear receptor PPARγ (374 candidates) from an initial set of ∼2.1 million molecules. Improved enrichment of true vs. false positives may be achieved by requiring a better ISE score cutoff or by performing docking. Those candidates can be purchased and tested experimentally to validate their activity. Further, we performed docking to CB2R structures and found lower statistical performance of the docking (“structure-based”) compared to ISE modeling (“ligand-based”). Therefore, ISE modeling may be a better starting point for molecular discovery than docking.

## 1 Introduction

The cannabinoid receptors (CBRs) consist of cannabinoid receptors 1 (CB1R) and 2 (CB2R), which are members of the lipid class A G protein-coupled receptors (GPCRs) family. The CBRs participate in many physiological processes, including mood regulation, cognitive function, neuroprotection, nociception, cell growth and proliferation, appetite, and lipid metabolism (Stasiulewicz et al., 2020). Both are expressed in the central nervous system (CNS) and in peripheral tissues. CB2Rs have lower expression levels than CB1Rs in the CNS and are primarily expressed in immune cells (Wu, 2019). Their different expression regions in the brain suggest a neuroprotective role of CB2R, avoiding CB1R mediated side-effects (Deng et al., 2015). Moreover, CB2R expression can be upregulated in the brain under some pathological conditions (e.g., addiction, inflammation, anxiety), suggesting CB2R involvement in various psychiatric and neurological disorders (Wu, 2019).

In the brain, CB2R is proposed as a potential target for attenuating inflammation associated with neurodegenerative diseases (Alzheimer’s disease (AD), Parkinson’s disease (PD), and others) (Cassano et al., 2017; Bie et al., 2018; Kelly et al., 2020; Mecha et al., 2020). Several selective CB2R agonists exhibited analgesic activity in preclinical models of acute inflammatory, chronic, and neuropathic pain (Murineddu et al., 2013; Soliman et al., 2021). Its role is also investigated in mental disorders like schizophrenia, depression, anxiety, and addictions (García-Gutiérrez et al., 2010; García-Gutiérrez and Manzanares, 2010; Ortega-Alvaro et al., 2011; ZX et al., 2011; Jordan and Xi, 2019; ME et al., 2019). Other potential therapeutic areas of CB2Rs were explored: anti-cancer (Guzmán, 2003; Fernández-Ruiz et al., 2007), epilepsy (Ji et al., 2021), osteoporosis (Idris et al., 2005; Rossi et al., 2011), atopic dermatitis (Maekawa et al., 2006), (NCT00697710), ischemia/reperfusion injury (Bátkai et al., 2007; Rajesh et al., 2007), atherosclerosis (Mach et al., 2008), gastrointestinal inflammation (Wright et al., 2008) and disorders of reproduction (Maccarrone, 2008).

In the past 2 decades, treating multifactorial illnesses, i.e., infections, cancer, and CNS disorders, shifted towards multitargeting (Csermely et al., 2005; Hopkins et al., 2006; Boran and Iyengar, 2010; L.; Bolognesi, 2013; Bolognesi and Cavalli, 2016; Zhou et al., 2019). Simultaneous modulation of multiple targets may have better efficacy and safety profile than single targeted drugs, and the number of multitargeting new molecular entities is increasing over the years (Ramsay et al., 2018). The design of multitargeting agents (MTAs) assigns desired therapeutic targets and avoids targets associated with side effects (“anti targets”). In principle, MTA can be a single compound or a combination of compounds, each directed to a different target (“cocktails” or as a co-formulated drug-device), and both are used in the clinic. Despite the highly significant therapeutic relevance of combinatorial therapy (Conway and Cohen, 2010; Morphy, 2010; Wright, 2010; Modi et al., 2011; Lu et al., 2012), single MTA has substantial advantages over combination therapy: 1) more predictable pharmacokinetic profile 2) avoiding drug-drug interactions 3) easier dose regimen and higher compliance 4) enabling to overcome mutations in relevant diseases such as cancer, viral and bacterial ailments 5) simultaneous presence of the molecule in tissues where it is expected to affect and 6) an easier regulatory process (Hopkins, 2008; Anighoro et al., 2014).

Targets from different protein superfamilies may challenge the design of such MTAs, lacking shared/similar ligands or common structural motifs, which are sometimes the cause of side-effects (Morphy et al., 2004). Therefore such different targets may be of more interest. Nevertheless, single MTAs have been discovered (Ryckmans et al., 2002; Natesan Murugesan et al., 2004; Omar et al., 2018).

The broad involvement of CB2R in various disorders makes it a valuable target for multitargeting therapies while combining its modulation with affecting other relevant proteins in each disease. Several studies proposed its combination with other targets such as acetylcholinesterase (AChE) and butyrylcholinesterase for AD (Gonzalez-Naranjo et al., 2013; Dolles et al., 2016, 2018; González-Naranjo et al., 2019). Suggestions were also raised to find dual CB2R/histone deacetylases and CB2R/σ receptor compounds for treating cancer and neurodegenerative diseases (Mangiatordi et al., 2020), and to develop multitargeting analgesics (Maione et al., 2013). Here we shall focus on several possibilities of multitargeting CB2R with other targets.

### 1.1 Combined Effects of CB2 and CB1 Receptors

The CBRs play a critical role in several human physiological and pathological conditions. However, the CNS side effects of CB1R ligands may limit the therapeutic use of such agents if they cross the Blood-Brain Barrier (BBB). That is the case of the CB1R inverse agonists Rimonabant and Taranabant (Moreira and Crippa, 2009; Martín-García et al., 2010). To overcome the central effects, peripheral CB1R antagonists were developed (Chorvat, 2013; El-Atawneh et al., 2019; Quarta and Cota, 2020). Another option is to develop pure antagonists (An et al., 2020; Stasiulewicz et al., 2020). Agonists of the CBRs may be used to treat anxiety (Stasiulewicz et al., 2020) or as analgesics, anti-inflammatory, neuroprotective and anti-emetic compounds (An et al., 2020). Peripheral CB1R antagonists combined with CB2R agonists may be used for treating liver diseases (Mallat et al., 2011) and diabetic complications (Gruden et al., 2016). This dual activity may be useful in treating obesity, abolishing diabetes-induced albuminuria, inflammation, tubular injury, and renal fibrosis (Barutta et al., 2017). Combining CB1R antagonism with CB2R agonism in the brain is shown to have a synergistic effect on reward processing (Gobira et al., 2019). Another option is to design selective CB2R agonists to benefit from their nociception and neuroinflammation role without psychoactive effects (Hollinshead et al., 2013; Verty et al., 2015; Poleszak et al., 2020). CB2R selective agonists are investigated to treat pain, inflammation, arthritis, addictions, cancer besides their neuroprotective role (An et al., 2020).

### 1.2 Combined Effects at CB2R, PPARγ, and 5-HT4R

CB2R could be targeted with other receptors to attenuate inflammation for several autoimmune and inflammatory conditions. The peroxisome proliferator-activated receptor (PPAR)-γ is a nuclear receptor that plays a crucial role in regulating lipid metabolism and glucose homeostasis. It associated with metabolic disorders, such as atherosclerosis, obesity, metabolic syndrome, dyslipidemias, type 2 diabetes, and cancer (Decara et al., 2020). PPARγ agonists have been shown to prevent inflammation, dermal fibrosis, and lipoatrophy in preclinical models of systemic sclerosis (SSc) (Wei et al., 2010). SSc is an orphan autoimmune multi-organic disease that affects the connective tissue. Dual CB2/PPARγ agonists such as VCE-004.8 and JBT-101 (Ajulemic acid, Lenabasum) have alleviated skin fibrosis and inflammation in SSc models (Rio et al., 2018; García-Martín et al., 2019). JBT-101 is in clinical trials for SSc (NCT03398837), dermatomyositis (NCT03813160), and cystic fibrosis (NCT02465450). Additionally, PPARγ agonists can suppress the pro-inflammatory cytokines associated with chronic diseases such as Inflammatory Bowel Disease (IBD).

IBD, including ulcerative colitis (UC) and Crohn’s disease (CD), has been considered one of the most prevalent GI diseases with accelerating incidence in newly industrialized countries. Yet it lacks effective drug targets and medications (Seyedian et al., 2019). As a lifelong disease, therapy aims to induce remission in the short term and maintain remission in the long term. New drugs have diverse mechanisms of action, targeting mainly the inflammation pathways. The current anti-inflammatory small molecules used to treat IBD are associated with several side effects (5-amino salicylate and its prodrugs such as Olsalazine and Balsalazide), with more severe toxicity (Azathioprine, Mercaptopurine, Methotrexate) or with known long term negative impacts of steroid hormones (glucocorticoids). Biological drugs are expensive, require more intensive medical attention in a clinic or at home (self-injections), and, in the case of TNFalpha antibodies, elicit resistance by immune system response (Torres et al., 2020). Although the mechanism by which PPARγ acts on the pathogenesis of IBD has not been clarified (Decara et al., 2020), natural and chemical PPARγ ligands have ameliorated the fibrotic process in preliminary clinical trials and experimental models of intestinal fibrosis (Vetuschi et al., 2018). Moreover, many studies showed the anti-inflammatory role of PPARγ activation in intestinal tissues in UC and CD (Decara et al., 2020).

Recent investigations suggest that serotonin (5-HT) can influence the development and severity of inflammation within the gut, particularly in the setting of IBD. 5-HT influences every major function inherent to the gut, including motility, secretion, blood flow, and sensation (Coates et al., 2017). Alterations in its receptor activity in disease conditions may result in many problematic symptoms, including abdominal pain, diarrhea, or constipation (Coates et al., 2017). The 5-HT4 receptor (5-HT4R) mediates enteric neuron survival and neurogenesis of adult mice (Liu et al., 2009). It promotes the reconstruction of an enteric neural circuit leading to the recovery of the defecation reflex in the distal gut (Matsuyoshi et al., 2010). 5-HT4R activation maintains motility in healthy colons of mice and guinea pigs and reduces inflammation in colons of mice with colitis (Spohn et al., 2016). PPARγ and 5-HT4R agonists may be combined with CB2R as a potential therapy for IBD (Turcotte et al., 2016). A peripheral CB2R agonist (Olorinab) reached phase II trials for abdominal pain in CD (NCT03155945) and irritable bowel syndrome (NCT04043455).

### 1.3 Multitargeting in Silico

Computational methods allow us to examine options for designing or discovering multitargeting candidates in a reliable, fast, and low-cost manner (Sliwoski et al., 2014; Zhang et al., 2017). Screening candidates for binding against several targets to find single MTA differs from designing compounds based on conjugated pharmacophores by merging/fusing/linking molecules (Morphy and Rankovic, 2005; Zhou et al., 2019), which could take longer to synthesize and might increase the molecular weight and affect the drug-likeness properties.

Our research combines ligand and structure-based methods. Our algorithm for solving complex combinatorial problems, the 'Iterative stochastic elimination algorithm’ (ISE) (Stern and Goldblum, 2014; El-Atawneh and Goldblum, 2017), has been applied in recent years to molecular discovery (Zatsepin et al., 2016; Da’adoosh et al., 2019; El-Atawneh et al., 2019), including one example of multitargeting modeling: modeling the properties of molecules that may be remotely loaded to nanoliposomes and the properties that enable them to be stable inside the nanoliposomes, in a biological fluid (Cern et al., 2017). Molecules that had high scores in both loading and stability models were chosen. For any discovery of MTAs, virtual screening (VS) by separate ligand-based models is performed in sequential order.

After finding top candidate ligands, it is helpful to examine the structural aspects, since our classifications are based on physicochemical properties and not on structural elements. Molecules with similar properties might have different structures and sizes. Thus, we dock the top candidates to the target protein if such a structure has been reported. Structures of CB2R were deposited recently in the Protein Data Bank (PDB), one with a bound antagonist (PDB code 5ZTY) (Li et al., 2019) and the other with an agonist (PDB code 6KPC) (Hua et al., 2020), which makes structure-based design feasible (Tuccinardi et al., 2006; Cichero et al., 2011). CB2R shares 44% sequence identity and 68% similarity with CB1R in the transmembrane regions (Munro et al., 1993). The antagonist-binding pockets in both receptors are quite distinct, while the agonist-binding pockets in CB1R and CB2R, including side-chain rotamers, of the key residues involved in ligands interactions are almost identical (Li et al., 2019; Hua et al., 2020), which might be the source of cross-reactivity between their ligands and difficulty in attaining selectivity. There are also CB1R and PPARγ structures, with agonists and antagonists in both. Yet, there is no published atomic-level structure of 5-HT4R, but ligand-based modeling for 5-HT4R with ISE is possible due to its many known ligands.

## 2 Methods

### 2.1 Data Sets

#### 2.1.1 Learning\Training Sets

Compounds with reported activity, agonists (EC_50_ values) and antagonists (k_i_ or IC_50_ values) at the different receptors were taken from the ChEMBL database (http://www.ebi.ac.uk/ChEMBLdb/) (Bento et al., 2014). Duplicates were removed based on their simplified molecular input line entry specification (SMILES notation). Molecules with undefined potency values, error comments, and a confidence score below seven (reported at ChEMBL) were excluded, as well as molecules that are active above 100 µM. The active molecules were diluted with random molecules assumed to be inactive (“decoys”) with a ratio of 1:100 (active: inactive) (Tropsha, 2010). Randoms were picked from the ZINC database (Sterling and Irwin, 2015), based on the “applicability domain” (APD) of the actives (Netzeva et al., 2005). The application of APD for picking randoms imposes to discover differences between active and inactive molecules with some basic similarities, thus making the task of classification more difficult. We apply APD by selecting random molecules for which the values of molecular weight (MW), calculated lipophilic character (clogP), hydrogen bond acceptors (HBA), and hydrogen bond donors (HBD) are within the average ± two standard deviations for these variables of the active molecules.

#### 2.1.2 Screening Set

The Enamine HTS Collection (Enamine HTS Collection 2021), consisting of 2,159,632 compounds was used for VS in both ligand and structure-based methods.

### 2.2 Datasets Preparation

All molecules were prepared by the “Molecular Database Wash” (v. 2011.10) (Molecular Operating Environment, 2021). This includes hydrogen adjustment, removing minor components, determining the protonation state, enumeration of ionization states, and tautomer forms. Mutagenic and reactive molecules (based on calculated descriptors by MOE) were removed from the learning sets.

### 2.3 Descriptors Calculation

The standard descriptors we calculated for building the models are the 2-dimensional (2D) descriptors by QuaSAR**-** MOE (v.2011.10) with 186 descriptors. The complete descriptors list is given at (http://www.cadaster.eu/sites/cadaster.eu/files/challenge/descr.htm). Descriptors with low variance (Smialowski et al., 2010), or highly correlated descriptors (Pearson correlation coefficient > 0.9), were excluded, using the Knime platform (v. 4.0.1) (Berthold et al., 2008) to exclude out of two highly correlated descriptors the one which has greater similarity to other descriptors. We have also tested the performance of 3D descriptors for CB2R (see results and discussion).

### 2.4 Activity Models Constructed by the Iterative Stochastic Elimination Algorithm

Our generic ISE algorithm has been applied to many problems related to drug discovery and has been presented in reviews, with details of the mathematical and statistical criteria to distinguish between two activities based on physicochemical properties (descriptors) of known active vs. inactive compounds (Stern and Goldblum, 2014; El-Atawneh and Goldblum, 2017). For each model, five cross-validations were performed (James et al., 2013), with 4 out of the five-folds producing the model, and the fifth fold was used as a test set. We include some of the main details of model construction and screening in Supplementary Data section 1.1.

### 2.5 Tanimoto Fingerprint Similarity

The “Atom-pair” fingerprints for the active molecules were generated using RDKit toolkit (RDKit, 2018) (in Knime platform v. 4.0.1) (Berthold et al., 2008). The “Tanimoto similarity coefficient” (Tc) for the fingerprints is based on the CDK toolkit.

### 2.6 Docking

The two structures of CB2R were downloaded from the PDB (5ZTY (Li et al., 2019) and 6KPC (Hua et al., 2020)), and prepared by the “Protein Preparation Wizard” (Schrödinger Suit 2019-3) (Madhavi Sastry et al., 2013). For 5ZTY, we allowed C-OH rotations of SER90, THR114, TYR190; for 6KPC, we allowed such rotations of TYR25, SER90, THR114, TYR190, and SER285 for the grid construction. Alanine (ALA) scan was performed to assign the critical residues in the binding site of the two structures for 23 residues detected by PDBsum (Laskowski, 2009). The screened molecules were prepared using “LigPrep” (Schrödinger Release, 2018), with default settings, except the chirality option that was set to “Generate all combinations” for the Enamine database (5,024,833 entries were generated). Molecular docking was performed with Glide HTVS and SP (Richard A. Friesner et al., 2006).

In the docking analysis, we examined the geometric character of binding by requiring the docked molecules to be in contact with residues that were found to be “hot spots” by performing a virtual ALA scan.

## 3 Results

### 3.1 Ligand-Based Approach

#### 3.1.1 Iterative Stochastic Elimination Algorithm Activity Models

We constructed several models for each target based on the relevant molecular activity reported by ChEMBL. There are molecules reported as partial agonists and inverse agonists for the CB2R (access date: January/2016), and those were excluded from the present study. Some models were constructed with a subset of highly active molecules (i.e., activity values less than 5 nM or 10 nM) from the larger set of reported activities. We choose the best-performing model based on Matthews Correlation Coefficient (MCC, Supplementary Data S1.1) (Matthews, 1975), Area under the ROC curve (AUC), and the Enrichment Factor (EF, Supplementary Data S1.1) (Table 1). Only ten molecules were reported with IC_50_ activity for 5-HT4R (access date: December/2017), so we used the reported K_i_ values for constructing the antagonist models (reported for 227 molecules). For PPARγ (access date: February/2018) and 5-HT4R agonist models, we built only one model based on the available data. The PPARγ antagonist models (access date: October/2021) have similar performance, and we chose the K_i_ model because it has a better EF value. All models have good mean MCC values > 0.65, AUC > 0.9, and EF values vary from 12 to 71 with a positive (> 0.0) index cutoff. The learning sets’ similarity is low for all chosen models (average Tc ≤ 0.5, Supplementary Table S1 **)**.

**TABLE 1:** Models of agonists and antagonists for the four receptors^a^.

	Model	# Actives	# Randoms	Top MCC	Mean MCC^c^	AUC	EF^d^	# Filters
CB2R agonists	Model 1 (Actives < 100 µM)	1254	100000	0.61	0.57	0.87	11 (38)	3911
	Model 2 (Actives < 5 nM)^b^	275	30000	0.73	0.70	0.90	17 (54)	2933
CB2R antagonists	Model 1 (IC_50_ values, Actives < 100 µM)	689	70000	0.64	0.57	0.85	18 (71)	1738
	Model 2 (IC_50_ values, Actives < 50 nM)	198	22000	0.73	0.69	0.91	8 (34)	3832
	Model 3 (K_i_ values, Actives < 100 µM)^b^	2437	200000	0.67	0.63	0.92	17 (56)	2747
CB1R agonists	Model 1 (Actives < 100 µM)	513	53000	0.66	0.62	0.89	11 (23)	3273
	Model 2 (Actives < 100 nM)	183	25000	0.8	0.77	0.90	11 (26)	2951
	Model 3 (Actives < 50 nM)^b^	127	13000	0.83	0.79	0.92	12 (27)	2509
CB1R antagonists	Model 1 (Actives < 100 µM)	973	93000	0.7	0.65	0.9	14 (33)	2231
	Model 2 (IC_50_ values, Actives < 10 nM)^b^	296	33000	0.78	0.75	0.92	25 (50)	1399
	Model 3 (K_i_ values, Actives < 10 nM)	332	35000	0.75	0.7	0.91	20 (65)	1960
PPARγ agonists	Model 1 (Actives < 10 nM)^b^	243	50000	0.91	0.89	0.96	62 (130)	3299
PPARγ antagonists	Model 1 (IC_50_ values, Actives < 10 nM)	194	20000	0.91	0.86	0.98	37 (74)	2677
	Model 2 (K_i_ values, Actives < 100 nM)^b^	168	17000	0.93	0.91	0.96	71 (98)	682
5-HT4R agonists	Model 1 (Actives < 100 µM)^b^	155	35000	0.94	0.92	0.98	37 (94)	3122
5-HT4R antagonists	Model 1 (K_i_ values, Actives < 100 µM)	227	50000	0.85	0.81	0.96	20 (61)	1035
	Model 2 (K_i_ values, Actives < 50 nM)^b^	148	35000	0.94	0.92	0.98	29 (52)	1475

a For each model, we present the number of active and random molecules used to generate the model, the top and average MCC of the filters, the AUC and EF values of the test set. Besides the number of the total filters generated by each model.

b The chosen models for VS.

c Mean MCC of the top 1000 filters.

d EF values above index cutoff = 0.7 are given in parenthesis.

# = number.

All constructed Models are presented in Table 1. The models used for screening are marked. Models constructed on the basis of active molecules with highest affinity (Nanmolar range) have better statistical parameters than those constructed on the basis of 100 µM activities, and were thus used for screening. That is the case of CB2R/CB1R/PPARγ agonists and antagonists, and 5HT4R antagonists. Only a single model of actives with lesser activity, of 5HT4R agonists, was used for screening. However the number of molecules with lesser affinity among the 155 used for modeling is small: only 5 molecules have EC_50_ values between 1 and 100 µM. Also, the 5HT4R model for agonists is the one with best statistical parameters compared to all other GPCR models for actives up to 100 µM.

##### 3.1.1.1 Performance of 3D Descriptors

Taking the learning set of the chosen 2D-based CB2R agonist model (Model 2- with 275 active molecules < 5 nM diluted with 30,000 randoms), we built 3D and the 2D/3D combined descriptors’ based models. The ISE agonist model based on 2D descriptors performed better than the 3D, and the 2D/3D combined descriptors by MCC, AUC, and EF (Supplementary Table S2). The 3D model has a lower mean MCC (0.5) and AUC (0.85) than the combined 2D/3D model.

### 3.2 Multitargeting Candidates

**FIGURE 1:**
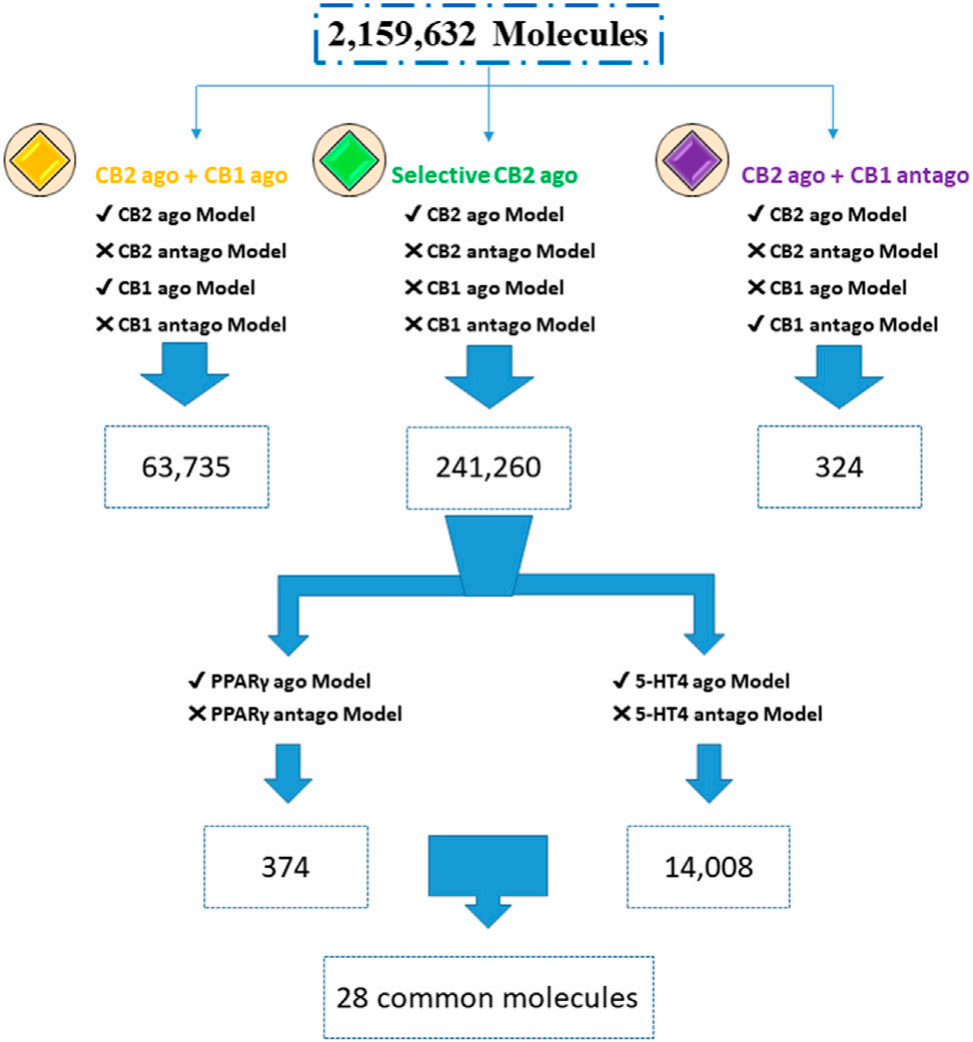
Screening for multitargeted candidates. Enamine database (2,159,632 compounds) was screened through agonist (ago) and antagonist (antago) ISE models. Numbers are of molecules with a positive index for models with a “✓” symbol, while failing to pass the models is marked by “X” (due to a negative index).

To find multitargeting candidates for the different indications, we performed hierarchical VS. First, focusing on the CBRs, we screened the Enamine database (DB) through the different CBR activity models, considering desired activity, i.e., of CB2R agonists, and the unwanted activity as anti targets. Molecules with a positive index pass the model, and those with a negative score are considered to fail. We found 241,260 CB2 selective agonists (about 11% of the dataset); those molecules passed the CB2R agonist model and did not pass the CB2R antagonist model. They also did not pass the CB1R agonist and antagonist models. Adding the CB1R agonists or antagonists to CB2 agonists, we found many less candidates (63,735 and 324, respectively), as shown in Figure 1. Raising the index cutoff above 0.0 reduces these numbers.

Looking for additional activities of the selective CB2R agonists, we screened those 241,260 candidates through the PPARγ and 5-HT4R agonist models (Figure 1). To avoid anti-targets we screened the same set by the antagonist models of PPARγ and 5-HT4R. This yielded 374 CB2R and PPARγ agonists, and 14,008 candidates for CB2R and 5-HT4R agonism with no antagonism at any of the three receptors. We found 28 candidate agonists for simultaneously hitting all the three targets of CB2R, PPARγ, and 5-HT4R. All the mentioned hit sets are internally diverse, as well as being diverse (by Tanimoto criteria) towards the actives used for model construction: comparisons yield a low average Tanimoto coefficient of Tc ≤ 0.4 (Supplementary Table S3).

#### 3.2.1 Common Substructures for the Multitargeting Hits

**FIGURE 2:**
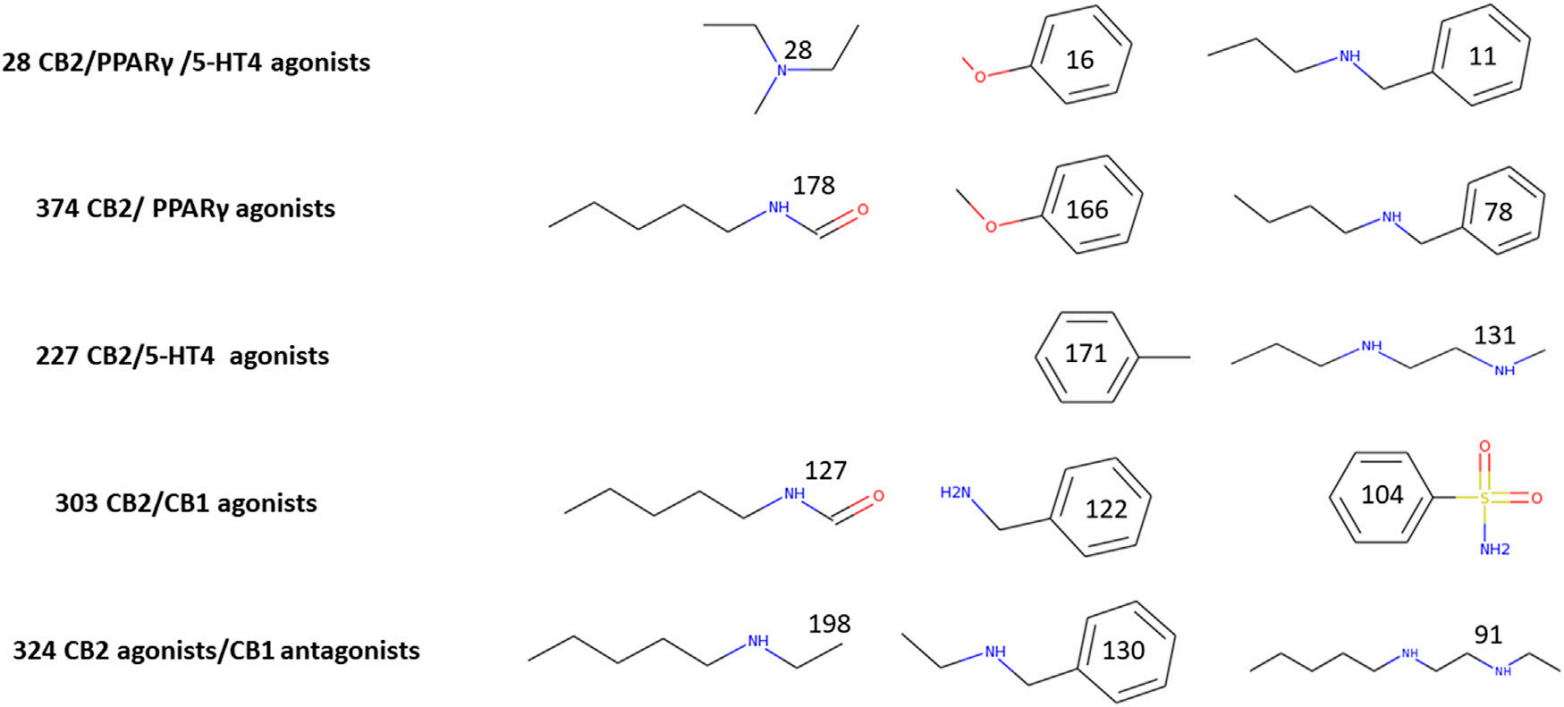
Major common multitargeted substructures. The numbers on each substructure indicate the number of molecules that include it. We chose 303 top candidates (with index score >0.7) for assigning substructures to CB2R/CB1R agonists and 227 top candidates (Index > 0.7) for the substructures of CB2/5-HT4 agonists. Other substructures were assigned for sets with an index > 0.0.

Common substructures could be used to explain why molecules are candidates for binding and activating different receptors. We examined that possibility for each multitargeting set. To perform that task, we used Canvas (v. 4.2.012, Schrödinger Suit 2019-4) to find the maximum common substructure. In Figure 2, we display the major common substructures for five different groups: agonists of all three receptors, CB2R/PPARγ, CB2R/5-HT4R as well as CB2R/CB1R agonists and CB2Ragonists/CB1R antagonists. A larger scope of common substructures is presented in Supplementary Figure S1.

Figure 2 presents major substructure elements of top multitargeted screened molecules. It is easy to detect some of the fragments which appear in more than 20% of each multitargeted group: tertiary and secondary amines, benzylamine, anisol, alkyl chains with amines or amide, and benzenesulfonamide. It is noteworthy that all the 28 CB2R/PPARγ/5-HT4R multitargeted candidates have a tertiary amine moiety, which is not abundant in either CB2R/PPARγ or CB2R/5-HT4R. Two fragments of CB2R/PPARγ—anisol and N-butylbenzylamine contribute to the triple multitargeting, while the only fragment of the CB2R/5-HT4R in the triple target is a phenyl ring. All three structures common to CB2R agonists/CB1R antagonists are secondary amines. Only a single secondary amine is among the main fragments of CB2R/CB1R agonists, and the two others are an aromatic sulfonamide and an amide of N-pentylamine.

### 3.3 Structure-Based Confirmation of CB2R Ligands

The structures of CB2R (6KPC (Hua et al., 2020) with an agonist and 5ZTY (Li et al., 2019) with an antagonist) have similar binding pockets and binding residues (Li et al., 2019; Hua et al., 2020) (Supplementary Table S4). Similarity is also observed between the CB2R and CB1R binding pockets (Li et al., 2019). This creates an obstacle to distinguishing between agonist and antagonist activity for the CB2R if we consider docking alone. We examined the binding residues in both structures by applying a virtual ALA scan (Schrödinger Suit 2019-3) (Madhavi Sastry et al., 2013) for 23 residues in the binding site (Supplementary Table S4). AM12033 (6KPC- CB2R agonist) has 19 interactions, mainly with hydrophobic and aromatic residues and 3 H-bonds, with LEU 182 and SER285. AM10257 (5ZTY- CB2R antagonist) has 16 interactions with no H-bonds (as shown in PDBsum (Laskowski, 2009)).

The calculated stability for the 23 residues (by virtual ALA scan) does not differ dramatically between 6KPC and 5ZTY. The considered contacts in the 6KPC agonist structure in order to suggest more successful docked ligands are: hydrogen bonding with LEU182 and SER285, and Van der Waals (VDW) interactions with the following: TYR25, PHE87, PHE91, PHE94, ILE110, PHE183, TYR190, LEU191, TRP194, LEU262, MET265, PHE281.

#### 3.3.1 Docking Validation

**FIGURE 3:**
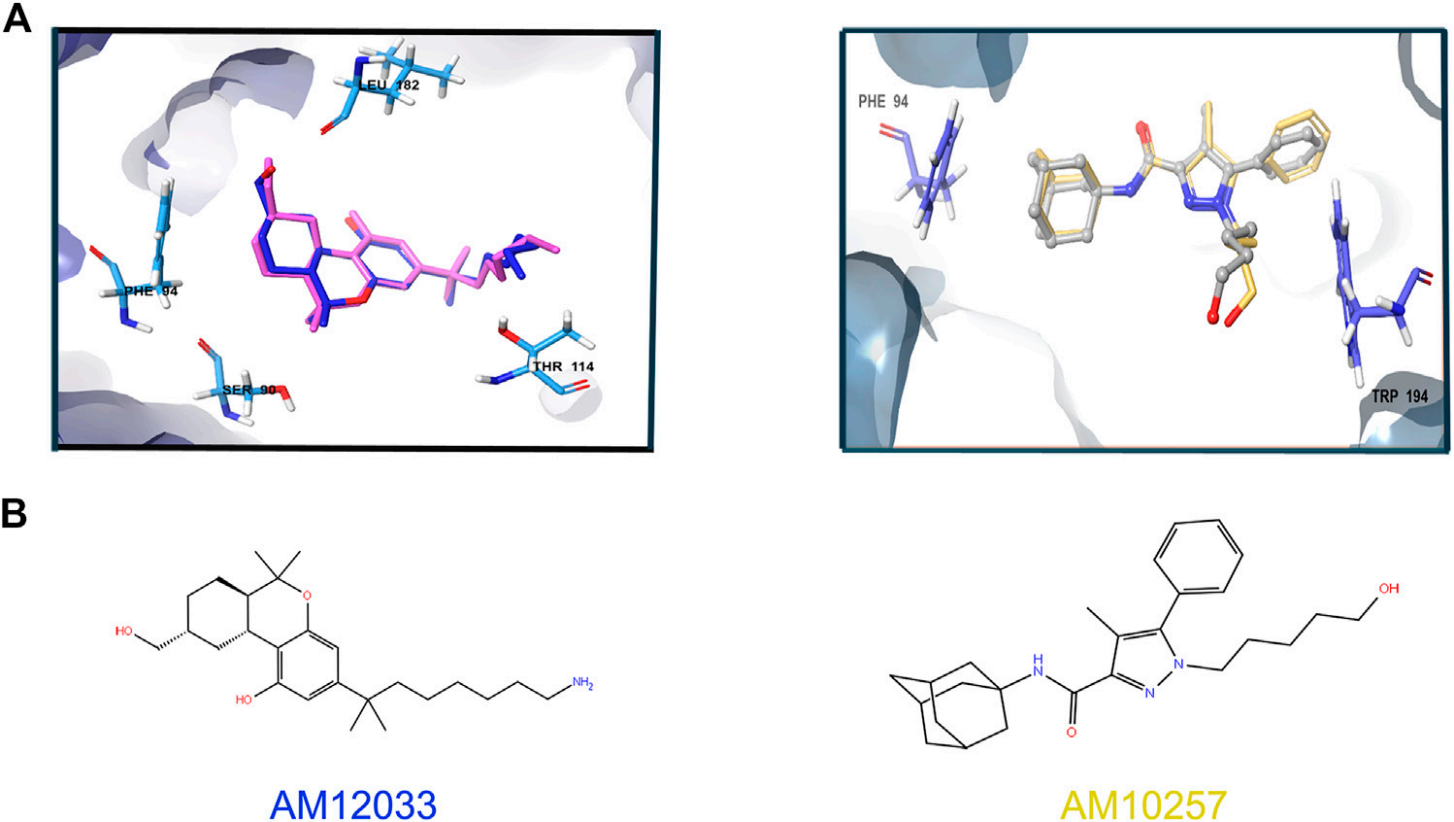
Superimposition of the redocked ligands at 6KPC and 5ZTY. **(A)** Left: relevant residues at 6KPC are shown in azure sticks (SER90, PHE94, LEU182, THR114 and LEU182). The redocked agonist (AM12033, docking score = −12.2 kcal/mol)—blue aligned with the original ligand (pink), with RMSD = 0.94. Right: relevant residues (PHE94 and TRP194) at 5ZTY are shown in blue sticks. The redocked antagonist (AM10257, docking score = −10.8 kcal/mol)—yellow aligned with the original ligand (gray), with RMSD = 1.5. **(B)** 2D representation of the agonist and antagonist ligands.

To choose one out of the two structures for detecting agonists and/or antagonists of CB2R, we constructed similar grids for the docking region in both structures, 6KPC and 5ZTY. We then redocked the ligands in both structures and performed cross-docking between the two. For 6KPC, the agonist, AM12033, got a better docking score (−12.2 kcal/mol) than the antagonist AM10257 (−8.7 kcal/mol). However, in 5ZTY, both agonist and antagonist got similar docking scores (−9.8 and −10.8 kcal/mol, respectively). The redocked positions of the agonist and antagonist are shown in Figure 3.

To further examine the binding of ligands to both structures, we docked overall 23 known ligands of CB2R and of CB1R with different selectivities (Supplementary Table S5) (An et al., 2020). Docking scores are not correlated with experimental K_i_ values (An et al., 2020) in Supplementary Table S5. Detailed interactions with binding site residues for the 19 ligands that passed docking to the 6KPC structure are listed in Supplementary Table S6. None of the interactions can be related to a specific activity. This is also seen in Supplementary Figure S2, where the best-docked ligand of each activity type is compared to the 6KPC ligand (AM12033). Finally, we screened the learning set of the CB2R agonist modeling (275 active molecules and 30,000 randoms), resulting in a very low AUC for docking to both 6KPC and 5ZTY: 0.45 and 0.44, respectively. The ISE model, however, got an AUC of 0.9. Due to the success in redocking an agonist, and the need for discovering agonists, we continued all docking experiments with 6KPC.

### 3.4 Virtual Screening: Ligand-Based vs. Structure-Based Methods

**FIGURE 4:**
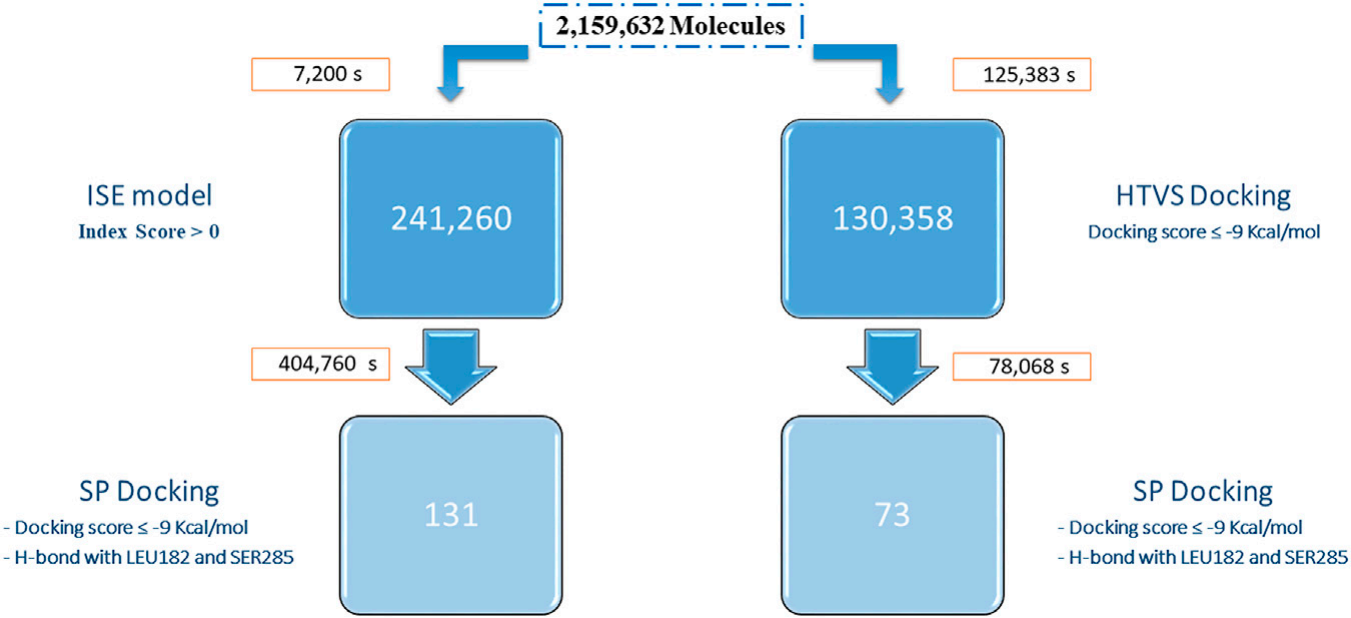
Workflows of VS of the Enamine database by ISE **(left)** and by docking **(right)**. The screening times (in seconds) and the number of candidates are indicated for each step. The SP docking for the ISE hits was performed for 857,546 entries (generated by ligprep from the 241,260 candidates). The docking protocol (HTVS, on the right) was performed for 5,026,503 entries (generated by ligprep). Only 130,358 molecules passed the score filtration, and those continued to SP docking.

We compared ligand (ISE) and structure-based (docking) methods by performing VS of the Enamine DB (2,159,632 compounds) for CB2R. ISE screening is extremely fast compared to docking (Figure 4). A positive index in screening by the CB2R agonist model was assigned to 241,260 molecules. We pick molecules with higher indexes and better EF values to improve the quality of our candidates, thus resulting in fewer molecules. For example, with a high index cutoff ≥ 0.7, 41,102 molecules pass, and the EF equals 54. That EF is only 17 at a lower index cutoff >0.0 (for 241,260 molecules). Docking was applied to the ISE candidates with a positive index: SP docking to the 6KPC structure found 238,718 molecules with docking scores of 6.6 to −12.8 kcal/mol. Filtration was based on docking scores ≤ −9 kcal/mol and hydrogen bonds with LEU182 and SER285, to a final set of 131 candidates.

Docking to CB2R was performed in two stages with the same 6KPC structure. First, HTVS docking was executed for the whole Enamine DB. The docked poses have a docking score range from 10.4 to −12.5 kcal/mol. Molecules with docking scores of less than −9 kcal/mol were further docked by the SP protocol (130,358 molecules). Most of these molecules (130,080) passed SP with a 5.7 to −12.9 kcal/mol docking score. By picking those with a score better than −9 kcal/mol and hydrogen bonds with LEU182 and SER285, only 73 molecules remain. Ten out of the 73 docking hits have positive ISE index scores. Only nine molecules are shared between the two SP screenings. Both sets are diverse from the known active CB2R agonists, and from each other (average Tc ∼0.3).

## 4 Discussion

The CBRs exert many physiological functions and are thus considered valuable therapeutic targets. CB2R, in particular, gains more attention due to its protective actions, involved in many pathological conditions such as cancer, CNS disorders, and a variety of disorders in the cardiovascular, gastrointestinal, and reproductive systems (Pacher and Mechoulam, 2011), while being devoid of psychoactive effects associated with the CB1R central activation. Finding single multitargeting agents (Morphy et al., 2004; Morphy and Rankovic, 2005; Zhang et al., 2017) for CB2R combined with other targets such as CB1R, PPARγ, and the 5-HT4R is not a trivial endeavor but one worth pursuing. Searching by virtual screening may suggest candidates in a shorter time than by *in vitro* screening and allows to test vast numbers of compounds. Our approach is to begin by constructing models for the binding or function of molecules at specific targets based on previously published results (“ligand-based” modeling). Our main tool for modeling is our ISE algorithm. The number of molecules for each model should not be less than a few dozens. Multitargeting requires to construct models for each of the relevant targets and anti-targets. If these models are of good quality, they may be used for VS, scoring, and sorting millions of molecules in a short time.

Here we present activity models built by the ISE algorithm for agonists and antagonists at each target. All models are statistically valid and should be useful (Table 1). The algorithm generates filters based on the ranges of physicochemical properties (computed) of known active molecules and randoms. Those filters are used for scoring by VS. It is noteworthy that the PPARγ and 5-HT4R models perform better than the models of CBRs. Their active sets are more similar (by Tc) than those of the CBRs, as shown in Supplementary Table S1. With an average Tc∼0.5, these sets of agonists may still be considered to be diverse. For VS, we use filters with top MCC values up to 20% below the maximal value or just the best 1,000 filters.

Choosing between 2D and 3D descriptors depends on the problem we want to solve. Even though 3D descriptors are more representative, they don’t yield better results, as have been studied in a large number and diverse range of applications over the past decades (Ekins et al., 2007). Some studies have shown that combining 2D and 3D molecular descriptors may improve models’ performance (Yera et al., 2011; Kombo et al., 2013). But for the CB2R agonist model, both the 3D-based and combined 2D/3D models have lower performance than the 2D-based model as shown in section 3.1.1.1.

Screening through ISE models was performed to find MTAs for several target combinations which reflect different indications (Figure 1). First, we screened through CBR models, which are involved in many pathological disorders. CB2R selective agonists have neuroprotective and anti-inflammatory effects (An et al., 2020). It is possible to reduce the number of molecules by increasing the cutoff index above 0.0. The higher that index, there will be less molecules to test further—but the enrichment factor, with more “true positives” will be greater. By performing SP docking of 241,260 molecules, subsequent to ISE modeling, we got 131 candidates (Figure 4). We got more candidates when combining CB2R agonists with CB1R agonist activity (63,735) rather than with CB1R antagonist activity 324) (Figure 1). That may be due to the high degree of structural similarity in the orthosteric binding pockets between agonist-bound CB2R and CB1R structures (Shahbazi et al., 2020).

Combining CB2R ligands that are active at CB1R might elicit central side effects associated with the CB1R. Therefore, it is important to limit CB1R activity to the periphery and avoid central activities, either agonistic or antagonistic. By applying criteria for peripheral action of CB1R ligands, it is possible to combine with CB2R ligands, particularly the combination of CB2R agonists/CB1R antagonists. Those candidates may be tested for multiple metabolic disorders, such as obesity and renal fibrosis (Barutta et al., 2017).

### 4.1 Some Implications of Ligand-Based Multitargeting

Multitargeting by ISE could be based on molecules with known activities on two or more targets. One publication mentions the construction of such a database, but it is not accessible (Chen et al., 2017). It is highly unlikely that enough molecules will be found to enable ISE modeling. Therefore, in the main spirit of ISE, each “variable” (in that case, a target, with many ligands as its “values”) requires separate model construction. Screening and scoring through any single model reduce the molecular library size by 10-fold or more. In HTS, it is common to discover 1 out of 1,000 molecules tested for activity. However, that is a real activity *in vitro*, while we only suggest candidates for *in vitro* testing, which may include false positives. Therefore their numbers are much larger.

As we add more targets and anti-targets, the number of candidates decreases: we found, among our ∼2.1 million screened molecules, only 374 candidates for combined (simultaneous) CB2R and PPARγ agonism, which may be tested for SSc (Wei et al., 2010), dermatomyositis, cystic fibrosis, and IBD (Decara et al., 2020). Adding 5-HT4R agonists reduces that number to 28, while CB2R and 5-HT4R agonists that could be valuable for IBD have 14,008 candidates. The much larger number of shared molecules that could hit CB2R and 5-HT4R (compared to sharing between CB2R and PPARγ) reflects the fact that both are aminergic GPCRs of the A family with 27% sequence similarity, as calculated by blastp (McGinnis and Madden, 2004), and may have a greater chance for ligand cross-reactivity (Yang et al., 2021). PPARγ belongs to a different family of cytoplasmic nuclear receptors. Moreover, only 60 molecules are shared between PPARγ and 5-HT4R agonists (without screening through CB2R models).

Screening by ISE models has already succeeded in achieving “scaffold hopping” (Zatsepin et al., 2016; Da’adoosh et al., 2019; El-Atawneh et al., 2019) due to the use of physicochemical properties rather than of structures. Even in those cases of greater similarity among the actives (agonists of PPARγ (0.52) and of 5-HT4R (0.5), Supplementary Table S1), the top screened candidates are varied among themselves, i.e., Tc = 0.4 for the 28 multitargeted agonists of CB2R/PPARγ/5-HT4R. That is also the case of screened molecules vs. actives in the learning sets (all results in Supplementary Table S3).

The main substructure elements presented in Figure 2 may help to understand how it is possible that a single molecule binds to different binding sites: the amine moieties—frequently two amines in a molecule—are singly charged, and the first protonation reduces the pKa of the other amine. Amine protonation prevails in four out of the five multitargeted sets, except for CB2R/CB1R agonists in which a negative charge on the oxygen of the amides may have a leading role. It is also clear from the difference between the coupling of CB2R agonists with either CB1R agonists or antagonists, that it is possible to separate between these multitarget pairs. It would still be impossible to suggest a synthesis of multitargeted compounds based on these major fragments, but it is easy to pick molecules that contain these fragments for each multitargeted alternative by requiring to include these substructures with their statistical weight as in Figure 2 or even better, as in Supplementary Figure S1. None of these moieties resemble the structures of known cannabinoid ligands (classical, non-classical, amino-alkylindoles, and those with the eicosanoid group).

### 4.2 The Impact of Structure-Based Modeling

Structures of CB2R have been recently deposited in the PDB (Li et al., 2019; Hua et al., 2020) and enable to perform structure-based studies—docking, pharmacophore, and molecular dynamics. The similarity between CB2R agonist/antagonist complexes and CB1R and CB2R structures make it challenging to design ligands with high selectivity (Hua et al., 2020). Docking is considered a time-consuming approach, as shown in Figure 4. Screening by docking has been shown to be much less reliable statistically than our ligand-based approach for CB2R agonism. Our ISE models screen molecules based on their properties and not on structural elements. That may result in top screened molecules having similar properties but different sizes and volumes, which may or may not be accommodated by the targets. Some of these molecules might not fit into binding sites and will be rejected. The results of our CB2R modeling confirm our preferable sequence of actions: ligand-based modeling should be followed by structure-based testing, which is better than structure-based docking alone.

Virtual ALA scan was used in this and other of our studies for picking “hot spots”—the main residues that contribute to the binding of smaller or larger ligands (i.e., including protein-protein interactions). Those “hot spots” determine the region of the grids for screening by docking and provide the initial geometric criteria that are applied prior to considering the docking scores. In ALA scan, we replace a larger side chain (of 18 amino acids, except for GLY and ALA) with a shorter one. We do not however apply any minimization or dynamics to that change, which positions a methyl group in the Cβ position, with tetrahedral angles vis-à-vis Cα, in place of a longer side chain, leaving some “void”. No other side chain position is modified around the virtually mutated one. This protocol is due to our wish to discover molecules that replace an existing ligand/protein with an exact similar conformation of side chains in the protein target, as in the PDB, in order to promote competition. That is clearly not the case with genetically mutated ALA scan. In that *in vitro* experiment, other side chains could change their conformations in the vicinity and more remote from the ALA mutated position. *In vitro* ALA scan may even change conformations of the main protein chain. Therefore, it is rewarding if mutagenesis studies support some of our results such as for PHE87, PHE91, PHE94, HIS95 (Li et al., 2019), and TRP194 (Zhang et al., 2011). TYR190 mutation to Ile resulted in a loss of ligand recognition and function (McAllister et al., 2002).

This is a theoretical study, which includes statistics (AUC, EF) that clarify what are the chances for discovering multitargeted actives. Naturally, the next step is to pick top candidates from each set for biochemical experiments. Our multitargeting results also suggest which multitargeting sets have a greater chance to be experimentally confirmed. Previously, we published our theoretical predictions and experimental validations of the binding of 8 molecules out of 15 predicted candidates (picked by ISE modeling from a library of 1.8 million) (El-Atawneh et al., 2019). Finally, only *in vitro* testing of candidates predicted by each method *in silico* will confirm or refute the VS results conducted by ISE and docking approaches.

## Data Availability

The original contributions presented in the study are included in the article/Supplementary Material, further inquiries can be directed to the corresponding author.
