# Supplementary material for: Candidate Therapeutics by Screening for Multitargeting Ligands: Combining the CB2 Receptor With CB1, PPARγ and 5-HT4 Receptors
Source: Front Pharmacol. 2022 Feb 28;13:812745. doi: 10.3389/fphar.2022.812745 (PMC8918518; doi:10.3389/fphar.2022.812745)
Supplement: Supplementary file 1 [file DataSheet1.PDF]

frontiers

Supplementary Material

1 Supplementary Data

1.1 Activity models constructed by the Iterative Stochastic Elimination (ISE) algorithm

ISE is a generic algorithm applied to find solutions for complex combinatorial problems. Its application to molecular discovery by classification requires to constructfilters based on calculated physicochemical properties (descriptors) of known actives diluted with random molecules (inactives). These filters are used for screening and scoring molecule libraries.

ISE searches for the best filters (each made up of a few descriptor ranges) that can distinguish between two activity classes. Each descriptor that is not binary is partitioned into a hundred discrete equally separated values, forming a large number (4950) of potential "ranges". The search begins by randomly picking one range for each calculated descriptor and screening the learning set through each range of any descriptor. Results are evaluated by counting the fraction of true positives (TP, actives which pass the range) out of the total actives, the fraction of false negative = actives that fail to pass (FN), as well as fractions of expected inactives that do not pass the range (TN, true negatives) and those inactives which do pass the range (False positives, FP). These four fractions (or percent) are introduced into the **Matthews Correlation Coefficient** (MCC- **Eq. 1**) (Matthews, 1975), which is particularly useful with unbalanced activity classes, i.e., as in our standard 1:100 ratio of actives vs. inactives. The best range of each descriptor is picked to generate the pool for producing random filters, with a single range representing each descriptor.

Filters are produced by random picking of 5 different ranges of descriptors from that pool. That process begins with a vast number of options (all possibilities of picking 5 ranges out of nearly 200), which ISE reduces by recording the effect of any single range on the quality of a large number of filters by considering MCC values only. Descriptors are then rejected if they consistently contribute to low MCC filters. The rejection is performed in iterations until we reach a step that enables to inspect all remaining filters exhaustively (once the remaining number is $<10^6$ combinations).

$$MCC = \frac{\mathrm{TP} * \mathrm{TN} - \mathrm{FP} * \mathrm{FN}}{\sqrt{(TP+FP)(TP+FN)(TN+FP)(TN+FN)}}$$

Equation 1. The MCC is a correlation coefficient between the observed and predicted binary classifications. True Positives-TP, True Negatives-TN, False Negatives-FN, False Positives-FP. It returns a value between −1 and +1. A coefficient of +1 represents a perfect prediction, 0 is no better than a random prediction, and −1 indicates total disagreement between prediction and observation or agreement with the reverse prediction.

The evaluation of the models is done by both MCC and the Area under the ROC curve (AUC) (Fawcett, 2006). Then we examine the Enrichment factor (EF) values (Pearlman and Charifson, 2001) (**Eq. 2)**.

$$Enrichment\ factor\ (EF) = \frac{TP/(TP + FP)}{(N_{Positives}/N_{Total})}$$

Equation 2. Enrichment factor equation, where $N_{Positives}$ is the total number of active compounds in the database and N_{Total} is all compounds in the set. EF is a measure of the ability of a classification model to identify TP compared to random picking so that the remaining portion of the database is richer in "hits" than the initial database

1.1.1 Screening and ranking molecules by ISE

Screening any set of molecules through the activity models (each is a set of very many filters) focuses on scoring each molecule due to its ability to pass or fail the passage of any filter. Each filter contributes a value-added for passing and subtracted for failure to pass. The scores from all filters are added and normalized by the number of filters, and the Index score of each molecule is thus between -1 and +1 (**Eq. 3**). The higher the index, the greater the probability of a molecule to be discovered experimentally as active.

$$Index\ score = \frac{\sum_{i=1}^{n} \delta_{positive}\, F_{positive(i)} - \delta_{negative} F_{negative(i)}}{n}$$

Equation 3. The Index Score. If the molecule complies with all filter ranges, it gets a positive weight ($\delta_{positive}$=1, $\delta_{negative}$=0). If it does not comply with one or more ranges of that filter, it gets a negative weight ($\delta_{positive}$=0, $\delta_{negative}$=1), the score is the sum of the weights of all the filters divided by the number of filters (n). The F-score refers to the harmonic mean of recall and precision, where recall refers to actual prediction accuracy, and precision defines the accuracy of a predicted class.

References:

Fawcett, T. (2006). An introduction to ROC analysis. *Pattern Recognit. Lett.* 27, 861–874. doi:10.1016/j.patrec.2005.10.010.

Matthews, B. W. (1975). Comparison of the predicted and observed secondary structure of T4 phage lysozyme. *Biochim. Biophys. Acta - Protein Struct.* 405, 442–451. doi:10.1016/0005-2795(75)90109-9.

Pearlman, D. A., and Charifson, P. S. (2001). Improved Scoring of Ligand−Protein Interactions Using OWFEG Free Energy Grids. *J. Med. Chem.* 44, 502–511. doi:10.1021/JM000375V.

2 Supplementary Figures and Tables

2.1 Supplementary Figures

A

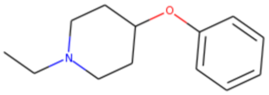

5 (18%)

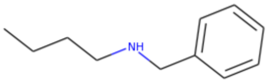

10 (35%)

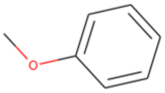

Anisol 16 (57%)

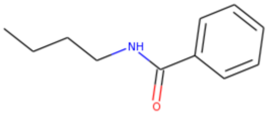

6 (21%)

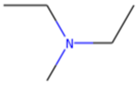

28 (100%)

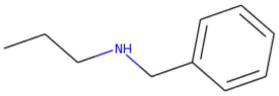

11 (39%)

B

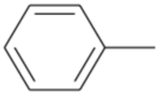

280 (75%)

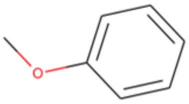

166 (44%)

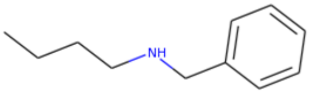

78 (21%)

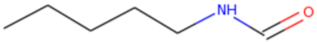

178 (48%)

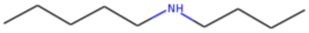

107 (28%)

C

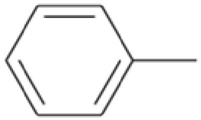

171 (75%)

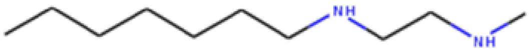

53 (23%)

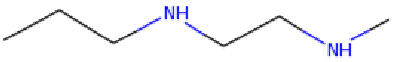

131 (58%)

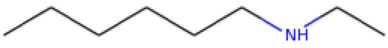

102(45%)

D

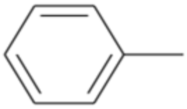

217 (72%)

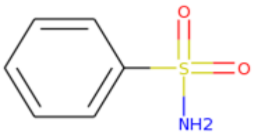

Benzenesulfonamide 104 (34%)

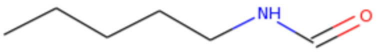

127 (42%)

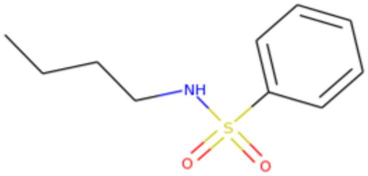

N-Butyl-Benzenesulfonamide 71 (23%)

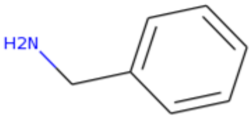

122 (40%)

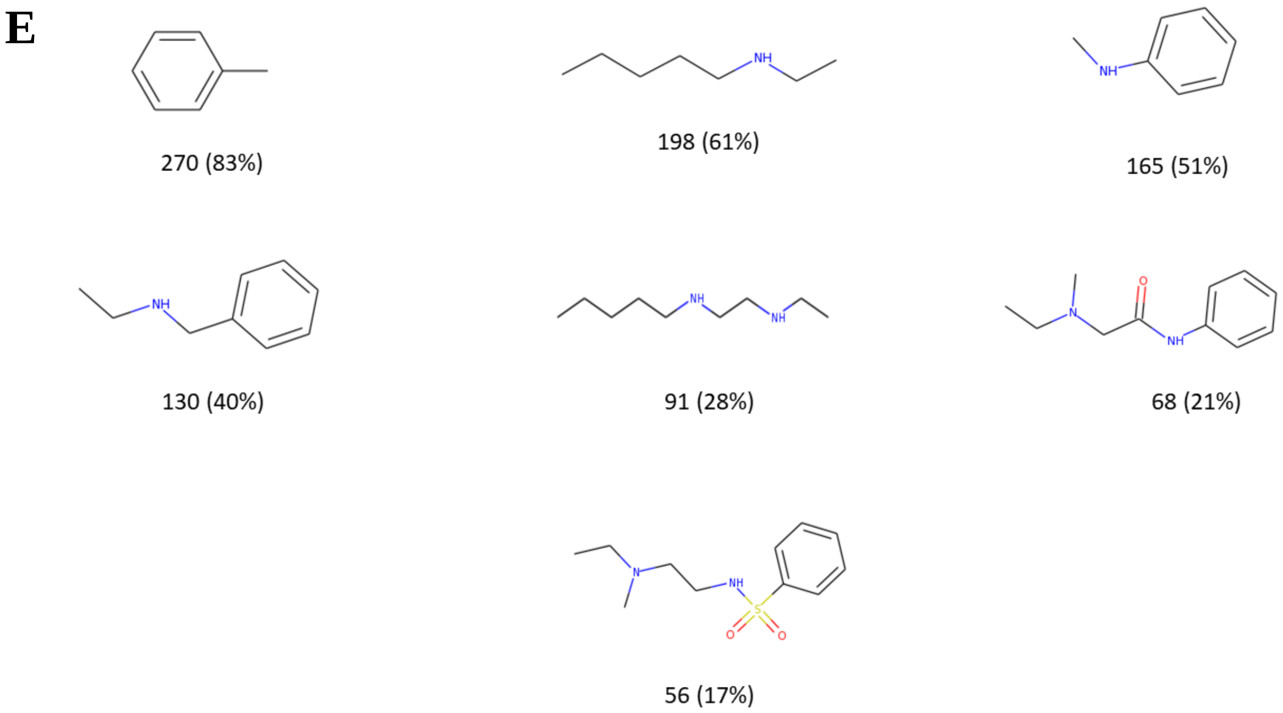

Figure S1. Common main substructures among multitargeting hits. (A) 28 CBR2/PPARγ/5-HT4R agonists, (B) 374 CB2R/PPARγ agonists, (C) 227 CB2R/5-HT4R agonists, (D) 303 CB2R/CB1R agonists, (E) 324 CB2R agonists/CB1R antagonists. The number of molecules including this substructure and percentage are indicated for each substructure.

A

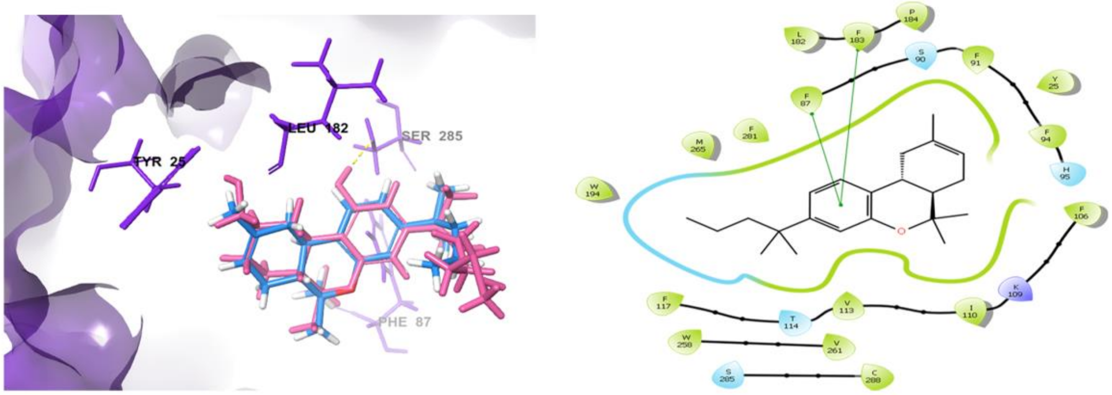
TYR 25
LEU 182
SER 285
PHE 87

B

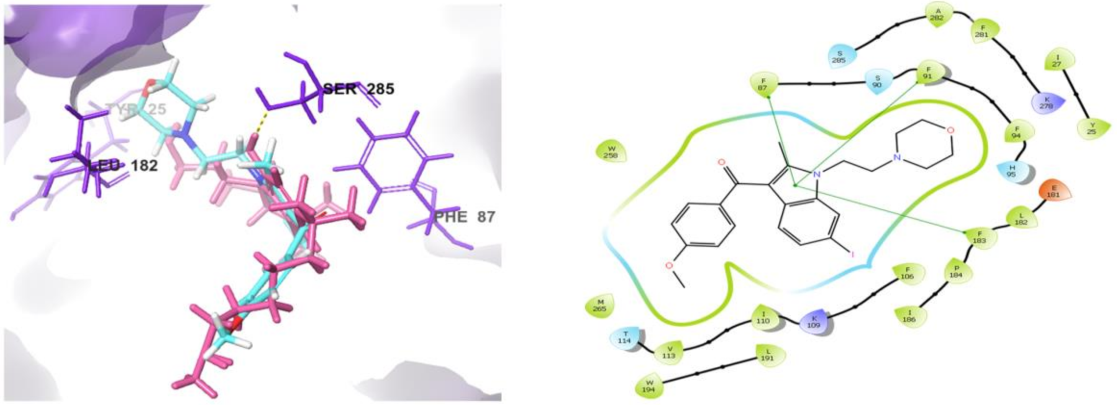
TYR 25
SER 285
LEU 182
PHE 87

C

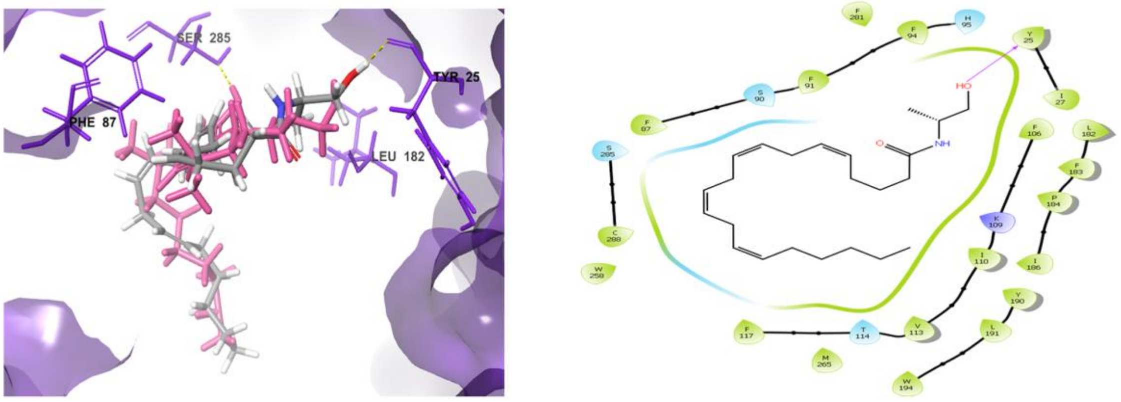
SER 285
TYR 25
PHE 87
LEU 182

D

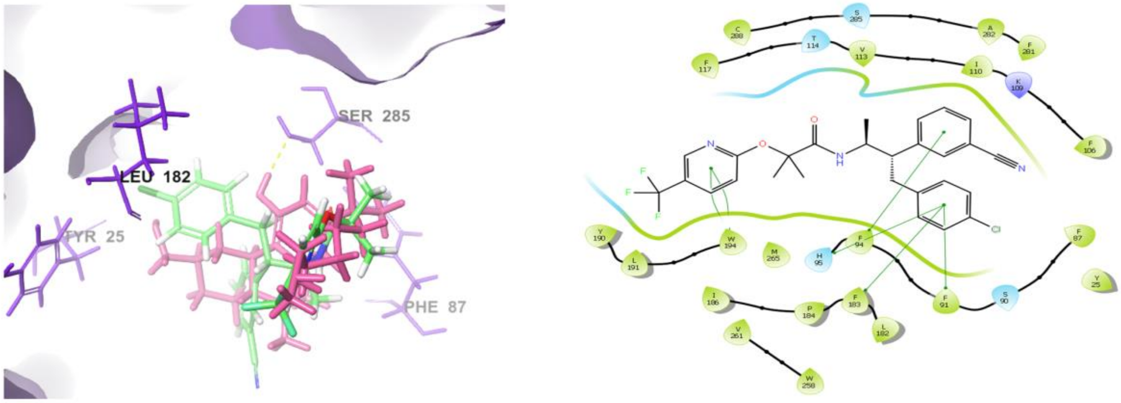

E

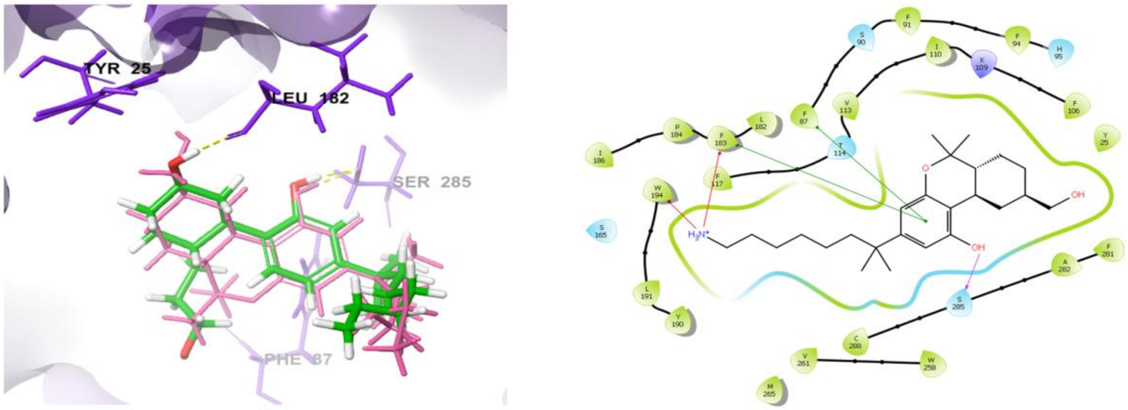

Figure S2. 2D and 3D representations of the best-docked ligands for the CB2R – 6KPC structure. For the 3D representation: we aligned the different ligands to the AM12033 (pink) in 6KPC structure, violet with residues shown as violet sticks. The different docked ligands from different activity types are shown as follows: (A) Selective CB2 agonist: JWH-133 - azure, (B) CB2-Selective antagonist/Inverse agonist: AM-630- cyan, (C) Selective CB1 agonist: Methanandamide (AM-356)- gray, (D) Selective CB1 antagonist: Taranabant- faded green, (E) Mixed CB1/CB2 agonist: CP-55,940 - green. Hydrogen bonds are represented as yellow dashed lines. For the 2D representation: violet arrows represent hydrogen bonds, green lines represent π-π interactions, and cation-π interactions are red lines as identified by Maestro 12.2 (Schrödinger Suite).
